# Supplementary material for: Long-Term Clinical Outcomes and Safety Analysis of Superficial Esophageal Cancer Patients Treated with Definitive or Adjuvant Radiotherapy
Source: Cancers (Basel). 2022 Jul 14;14(14):3423. doi: 10.3390/cancers14143423 (PMC9322572; doi:10.3390/cancers14143423)
Supplement: Supplementary file 1 [file cancers-14-03423-s001.zip › cancers-1773588-supplementary.pdf]

Supplementary Table S1. Summary of previous studies discussed in the current study

Year/First Author	Ref.	No. Patients	Stage/No. Patients	Methods/No. Patients	Result
2004/Wang GQ	[1]	N = 420 (ESCC)	Tis/N = 76<br>T1a/N = 126<br>T1b/N = 218	Esophagectomy/N= 420	5-year OS:<br>86.1%
2014/Merkow RP	[4]	N = 5390<br>4819(Adenocarcinoma)<br>71(ESCC)	T1a/N = 2911<br>T1b/N = 2479	ER/N = 1428<br>Esophagectomy/N = 3962	5-year OS:<br>26.5% vs. 87.6%
2018/Min YW	[5]	N = 240 (ESCC)	T1a/N = 182<br>T1b/N = 58	ER/N = 120<br>Esophagectomy/N = 120	5-year OS:<br>93.9% vs. 91.2%
2013/Yamashina T	[9]	N = 402 (ESCC)	T1a/N = 350<br>T1b/N = 52	ER/N = 402	5-year OS:<br>70.8% (T1b)
2020/Zhang Y	[10]	N = 70(ESCC)	T1a/N = 70	ER/ N = 35<br>ER+RT/ N = 35	3-year OS:<br>100% vs. 100%
2012/Motoori M	[12]	N = 173 (ESCC)	T1b/N = 173	Esophagectomy/N = 102<br>dCRT/N = 71	3-year OS:<br>87.0% vs. 77.8%<br>5-year OS:<br>77.7% vs.68.6%
2021/Jo YY	[13]	N = 282 (ESCC)	T1b/N = 282	Esophagectomy/N = 238<br>dCRT/N = 44	5-year OS:<br>75.8% vs. 68.8%
2021/Kato K	[14]	N = 368 (ESCC)	T1b/N = 368	Esophagectomy/N = 209<br>dCRT/N = 159	5-year OS:<br>86.5% vs. 85.5%
2021/Haneda R	[15]	N = 68 (ESCC)	T1b/N = 68	Esophagectomy/N =39<br>d CRT/N = 29	5-year OS:<br>92.9% vs. 77.8%
2021/Yang X	[16]	N = 31 (ESCC = 30)	T1a/N = 5	ER+ RT/N = 30	5-year OS:

		(Adenosquamous carcinoma=1)	T1b/N = 26		68.5%
2019/Tanaka T	[21]	N = 52 (ESCC)	T1b/N = 52	ER + Esophagectomy /N = 19<br>ER + CCRT/N =33	5-year OS:<br>89.5% vs. 80.3%
2021/Ikawa T	[22]	N = 96 (ESCC)	T1a/N = 32<br>T1/ N = 64	ER + CCRT/N = (96)	5-year OS:<br>82.4%
2021/Uchinami Y	[23]	N = 71 (ESCC)	T1a/N = 6<br>T1b/N = 65	RT or CRT/N =26<br>ER + CRT/N =45	5-year OS:<br>64.0%
2018/Yoshimizu S	[26]	N = 64 (ESCC)	T1b/N = 64	ER - CRT/N = 21<br>dCRT / N = 43	5-year OS:<br>85.1% vs. 79.1%
2018/Suzuki G	[27]	N = 32 (ESCC)	T1b/N = 32	ESD + Esophagectomy/N = 16<br>ESD + CRT / N = 16	2-year OS:<br>100% vs. 100%
2015/Kawaguchi	[30]	N = 47(ESCC)	T1a/N = 5<br>T1b/N = 65	ESD + CRT / N =16<br>dCRT/N =31	3-year OS:<br>90.0% vs. 63.2%%

Abbreviations: ESCC—esophageal squamous cell carcinoma; ESD—endoscopic submucosal dissection; ER—endoscopic resection; OS—overall survival; RT—radiotherapy; CCRT—concurrent chemoradiotherapy; CRT—chemoradiotherapy; dCRT—definitive chemoradiotherapy.
